# Prediction of properties of boron $\alpha$-icosahedral nanosheet by bond-addictive ${\mathbb {M}}$-polynomial

**DOI:** 10.1038/s41598-024-51642-2

**Published:** 2024-01-12

**Authors:** D. Antony Xavier, K. Julietraja, Ammar Alsinai, S. Akhila

**Affiliations:** 1 Department of Mathematics, Loyola College (Affiliated to the University of Madras), Chennai, India; 2 Department of Mathematics, School of Engineering, Presidency University, Bengaluru, 560064 India; 3 Department of Mathematics, Ibb university, Ibb, Yemen; 4 Department of Mathematics, Kuvempu University, Shivamogga, Karnataka 577451 India

**Keywords:** Bone, Orthopaedics

## Abstract

Nanosheets with boron elements have excellent characteristics which makes the boron polymorphs unique and super hard. A boron $\alpha$-icosahedral nanosheet in crystalline form has superconductivity and thermal electronic properties. In theoretical chemistry and QSPR/QSAR study, a topological descriptor is an important analytical tool. It helps to analyse the structure and its properties and also correlates the with numerical expressions. The valence-based M-polynomial provides quantitative measures of molecular properties based on their geometric, electrostatic, and quantum chemical characteristics. In this article, the QSPR/QSAR analysis is performed for this nanosheet and the analytical expressions are validated with original synthesized data, and received excellent correlation values of 0.9835 and 0.9932. The mathematical expression of the structure is analysed and the indices are compared graphically and numerically.

## Introduction

Boron is an interesting and complex element, many aspects of which are still to be explored. The properties of boron are found between metals and insulators. While boron has only three valence electrons, which would favor metallicity, they are localized enough to produce insulating states. However, pressure, temperature, and impurities can easily shift this subtle balance between metallic and insulating states. Pure boron is one of the best alternatives to carbon fullerenes (CFs) and nanotubes (CNTs), which exhibit superior properties, in the form of novel solids and nanostructures, such as quasiplanar clusters, quasi-crystals, nanosheets, nanoribbons, nano chains, and nanotubes^1^. Besides being the only non-metal element in Group III, boron is unique in its structural complexity and has exceptional chemical and physical properties, including low densities, high melting points, and high hardness^2^. Initially, Boron exists in three crystalline forms, $\alpha -B_{12}$, $\beta -B_{106}$ and $\gamma -B_{28}$^3^. Later different forms of boron crystalline have been synthesized, such as $\alpha$- rhombohedral, $\beta$-rhombohedral, tetragonal, $\gamma$-orthorhombic, and $\alpha$-Ga type. In addition, there are amorphous phases and nanosized structures^4^. One of them is the $B_{12}$ icosahedral that is linked together by “inter-icosahedral covalent units” or “chains”^5^. The boron-rich ceramics based on icosahedral are second only to diamonds as hard materials. When compared to diamond-based materials, this class of ceramics offer low density, better thermal and chemical resistance, and ease of mass production.

**Figure 1:**
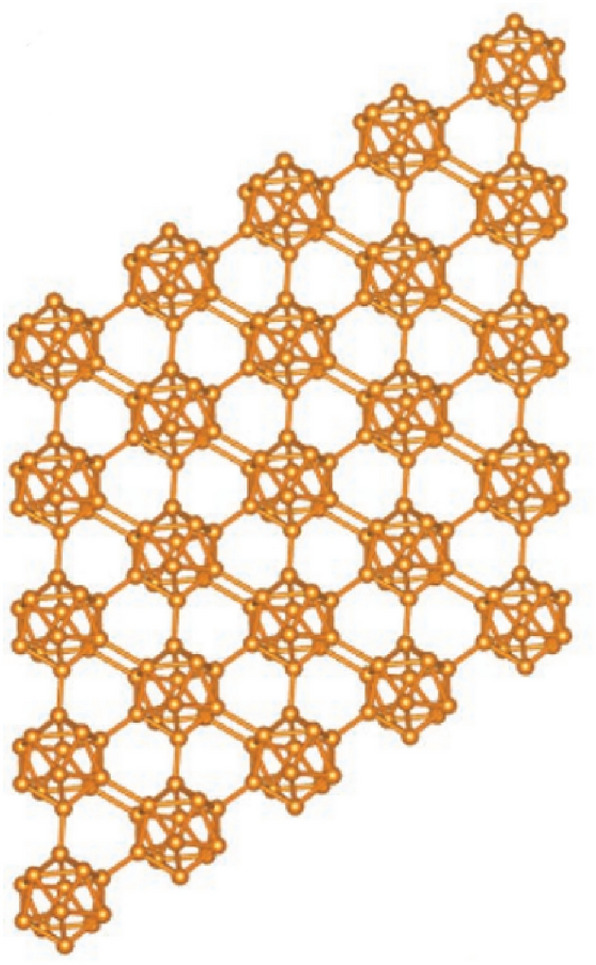
Crystal structure of boron $\alpha$-icosahedral nanosheet.

In boron $\alpha$-icosahedral nanosheet, each crystal contains an icosahedron molecule of $B_{12}$, which is linked to form a three-dimensional network^1^ as shown in Fig. 1. A regular icosahedron has 12 vertices, 30 edges and 20 faces. The icosahedral boron sheet, $B_{12}$ and $B_{20}$ have been proposed in recent years with special properties. Kah et al.^6^ proposed many icosahedral nanosheets based on $B_{12}$ clusters, and Zhou et al.^7^ presented an antiferromagnetic metallic $B_{20}$ sheet. Higashi et al.^8^ investigated the first 2D icosahedral $B_{12}$ networks. The icosahedral nanosheet bonding is complex and was well explained by Emin^9^. The boron allotropes attract major material researchers since they exhibit properties like thermal conductivity, hardness, and neutron scattering length^10^. The novel icosahedral structures exhibit interesting chemical bonding and electronic properties and are structurally and energetically stable. Additionally, these $\alpha$-icosahedral nanosheets, which are a gapless system, exhibit semiconducting properties, suggesting an application in nanoelectronics and computer chips. is a good choice, In industrial semiconductor applications like solar cells with high solar light conversion efficiency, the icosahedal boron nanosheet is a prominent component^11^.

In a molecular graph, each edge of a molecule corresponds to a chemical bond between atoms, while each vertex and degree denotes an atom and valence of the atom. In order to characterize the structural features of these molecules, several theoretical tools are employed. A topological index can be used to model relationships between chemical structures and their corresponding biochemical and physicochemical activities^12,13^. Large combinatorial chemical libraries are required to compute the physicochemical properties of a structure. These include novel development methods such as topological structural descriptors, combinatorial quantum chemistry tools for functional group analysis, shape-activity relations, and topological attributes of electron densities, etc. The degree-based topological indexes are used extensively in network science for investigating networks, in which the indexes are calculated based on the degrees of the graph. A breakthrough was made in degree-based indices by Deutsch and Klav$\breve{z}$ar^14^, introducing the M-polynomial. Readers can refer to^15–18^ for recent work in M-polynomial and topological indices.

Boron $\alpha$-icosahedral nanosheets are grabbing immense attention due to their numerous applications in emerging technologies. Thus, understanding the properties of these structures is imperative for industrial applications. In this paper, the degree-based structure analysis of $\alpha$-icosahedral nanosheet is performed using M-polynomial. The analytical expressions for some prominent indices are evaluated and their graphical representations are plotted using the numerical values of these indices and compared. The shear modulus and Young’s modulus of the icosahedral nanosheet are compared against its structural parameters, which helps to predict the properties of numerous additional boron allotropes.

## Computational techniques

**Figure 2:**
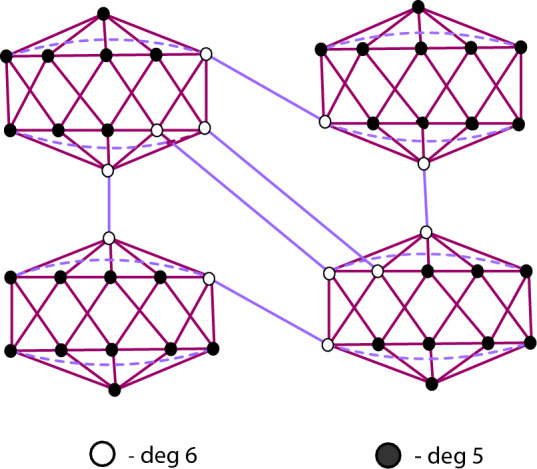
Degree of boron $\alpha$-icosahedral nanosheet.

A chemical compound can be modeled as a simple graph, $\chi$ with vertex and edge sets, ${\mathcal {V}}(\chi )$ and ${\mathcal {E}}(\chi )$ respectively. The valency of an atom is denoted by $\texttt{d}_\mu$ of the vertex $\mu \in {\mathcal {V}}(\chi )$, whereas the maximum degree over all the vertices of $\chi$ is denoted by $\Psi$. The degree of the vertex of boron $\alpha$-icosahedral nanosheet is illustrated in Fig. 2. The set are consider, ${{\mathfrak {D}}}= \{({\mathfrak {k,h}} \in \mathbb {N\times N})| 1\le {{\mathfrak {k}}}\le {{\mathfrak {h}}}\le \Psi \}$. We denote $\texttt{d}_{{\mathfrak {k,h}}}=|\{\mu \eta \in {\mathcal {E}}(\chi )|\texttt{d}_\mu ={{\mathfrak {k}}}$ and $\texttt{d}_\eta ={{\mathfrak {h}}}\}|$. The ${\mathbb {M}}$-polynomial^14^ for simple connected graph, $\chi$ is defined by

1 $$\begin{aligned} {\mathbb {M}}(\chi ;{\mathfrak {y,z}})=\sum _{{{\mathfrak {k}}}\le {{\mathfrak {h}}}}\texttt{m}_{{\mathfrak {kh}}}(\chi ){{\mathfrak {y}}}^{{\mathfrak {k}}}{\mathfrak {z}}^{{\mathfrak {h}}} \end{aligned}$$

where $\texttt{m}_{\mathfrak {kh}}(\chi )$ be the total number of edges $\mu \eta \in {\mathcal {E}}(\chi )$ such that $\{\texttt{d}_\mu ,\texttt{d}_\eta \}=\{{\mathfrak {k,h}}\}$. The bond additive is the function from $\chi$ into ${\mathbb {R}}$ specified as real numbers $\beta _{{\mathfrak {k,h}}}$, $({\mathfrak {k,h}})\in {{\mathfrak {D}}}$ induced by $\beta (\chi )= \sum _{({\mathfrak {k,h}})\in {{\mathfrak {D}}}}\texttt{d}_{{\mathfrak {kh}}}\beta _{{\mathfrak {kh}}}$. The degree-based structural descriptors for $\chi$, where $\texttt{f}(\texttt{d}_\mu , \texttt{d}_\eta )$ is the function of degree based indices is depicted as

$$\begin{aligned} \Lambda (\chi )=\displaystyle \sum _{\mu \eta \in {{\mathfrak {D}}}}\texttt{f}(\texttt{d}_\mu , \texttt{d}_\eta ) \end{aligned}$$

A brief discussion of bond additive degree-based indices is given below regarding the above-specified real numbers, $\beta _{{\mathfrak {kh}}}$. First degree-based structure descriptors were studied^19^ and developed^20^ with the Zagreb index, $M_1(\chi )$ defined by $\beta _{{\mathfrak {kh}}}= {\mathfrak {k+h}}$ based on the square root of the vertex degrees to analyze the influence of total electron energy on structure. The next analogous of Zagreb index is *second Zagreb index*, $M_2(\chi )$ represented as $\beta _{{\mathfrak {kh}}}= {\mathfrak {kh}}$. These indices help in analyzing the complexity of the molecular system and increase with extent branching of the carbon skeleton. The other analogous of Zagreb index are *augumented Zagreb index* , $AZ(\chi )$^21^ and *hyper Zagreb index*, $HM(\chi )$^22^ is defined by $\beta _{{\mathfrak {kh}}}= \big (\frac{{\mathfrak {kh}}}{{\mathfrak {k+h}}-2}\big )^3$ and $\beta _{{\mathfrak {kh}}}= ({\mathfrak {k+h}})^2$ respectively. These indices are used to analyze new drugs’ molecular structures and to understand their biological and chemical properties. Based on the inverse value of vertex degree, the other invariant of Zagreb index, *modified Zagreb*, $M_2^m(\chi )$^23^ defined by $\beta _{{\mathfrak {kh}}}= \frac{1}{{\mathfrak {kh}}}$ is evolved. Several studies have demonstrated that the augmented Zagreb index can predict the temperature at which octanes and heptanes form. These variants of Zagreb indices can be used for determining the isomerism of ZE, chirality, heat formation, and heterogeneity of hetero systems.

Based on the degrees of the end vertices of $\chi$, several methods have been proposed to examine the branching properties of alkanes. In 1975 Milan Randić^24^ developed the topological index of graph, $\chi$ under the label “molecular connectivity index” in the description $R_{-1}$ and $R_{-1/2}$. A *general Randić index*, $R_{{\mathfrak {d}}}(\chi )$ latterly developed by Bollobas and Erdos^25^ by substituting $R_{-1}$ and $R_{-1/2}$ with a real integer ${{\mathfrak {d}}}$ is defined as $\beta _{{\mathfrak {kh}}}= ({\mathfrak {kh}})^{{\mathfrak {d}}}$. The other variant of randić index are * reciprocal randić*, $RR_{{\mathfrak {d}}}(\chi )$^26^ and *harmonic index*, $H(\chi )$^27^ are represented as $\beta _{{\mathfrak {kh}}}= \frac{1}{({\mathfrak {kh}})^{{\mathfrak {d}}}}$ and $\beta _{{\mathfrak {kh}}}= \frac{2}{{\mathfrak {k+h}}}$. Graph eigenvalues were analyzed by Favaron et al.^28^ in relation to harmonic indices. A correlation has been demonstrated between these variants of randic index and various physicochemical properties of alkanes, including the formation of enthalpies, surface areas, vapor pressure, boiling points, Kovats constants, and so on^29^.

*Symmetric division degree index*, $SSD(\chi )$^30^ is a great tool for predicting polychlorobiphenyl surfaces is defined by $\beta _{{\mathfrak {kh}}}= \frac{{{\mathfrak {k}}}}{{{\mathfrak {h}}}}+\frac{{{\mathfrak {h}}}}{{{\mathfrak {k}}}}$ or $\beta _{{\mathfrak {kh}}}= \frac{{\mathfrak {k^2+h^2}}}{{\mathfrak {kh}}}$. *Forgotten index*, $F(\chi )$^31^, which greatly enhances the physicochemical prediction of the First Zagreb index, and it is defined as $\beta _{{\mathfrak {kh}}}= {\mathfrak {k^2+h^2}}$. An important tool for estimating octane isomer surface area is the *inverse sum index* $I(\chi )$^32^ defined as $\beta _{{\mathfrak {kh}}}= \frac{{\mathfrak {kh}}}{{\mathfrak {k+h}}}$. And *sigma index*, $\sigma (\chi )$ is given by $\beta _{{\mathfrak {kh}}}= ({\mathfrak {k-h}})^2$. By analyzing the above discussion, it is evident that the bond additive degree is a significant aspect to investigate the physicochemical properties of molecular structures. Table 1 outlines the formulations for the M-polynomial method.

**Table 1:** The derivation of vertex-degree ${\mathbb {M}}$-polynomials.

Topological indices	$\texttt{f}({\mathfrak {y,z}})$	Derivation from ${\mathbb {M}}(\chi )$
$M_1(\chi )$	${\mathfrak {y+z}}$	$(D_{{\mathfrak {y}}}+D_{{\mathfrak {z}}})({\mathbb {M}}(\chi ); {\mathfrak {y,z}})|_{{\mathfrak {y=z=1}}}$
$M_2(\chi )$	${\mathfrak {yz}}$	$(D_{{\mathfrak {y}}}D_{{\mathfrak {z}}})({\mathbb {M}}(\chi ); {\mathfrak {y,z}})|_{{\mathfrak {y=z=1}}}$
$M_2^m(\chi )$	$\frac{1}{{\mathfrak {yz}}}$	$(S_{{\mathfrak {y}}}S_{{\mathfrak {z}}})({\mathbb {M}}(\chi ); {\mathfrak {y,z}})|_{{\mathfrak {y=z=1}}}$
$A(\chi )$	$(\frac{{\mathfrak {yz}}}{{\mathfrak {y+z-2}}})^3$	$(S_{{\mathfrak {y}}}^3Q_{-2}D_{{\mathfrak {y}}}^3D_{{\mathfrak {z}}}^3)({\mathbb {M}}(\chi ); {\mathfrak {y,z}})|_{{\mathfrak {y=z=1}}}$
$R_{{\mathfrak {d}}}(\chi )$	$({\mathfrak {yz}})^{{\mathfrak {d}}}$	$(D_{{\mathfrak {y}}}^{{\mathfrak {d}}}+D_{{\mathfrak {z}}}^{{\mathfrak {d}}})({\mathbb {M}}(\chi ); {\mathfrak {y,z}})|_{{\mathfrak {y=z=1}}}$
$RR_{{\mathfrak {d}}}(\chi )$	$\big (\frac{1}{{\mathfrak {yz}}}\big )^{{\mathfrak {d}}}$	$S_{{\mathfrak {y}}}^{{\mathfrak {d}}} S_{{\mathfrak {z}}}^{{\mathfrak {d}}}(D_{{\mathfrak {y}}}+D_{{\mathfrak {z}}})({\mathbb {M}}(\chi ); {\mathfrak {y,z}})|_{{\mathfrak {y=z=1}}}$
$H(\chi )$	$\frac{2}{{\mathfrak {y+z}}}$	$2S_{{\mathfrak {y}}}J({\mathbb {M}}(\chi ); {\mathfrak {y,z}})|_{{\mathfrak {y=z=1}}}$
$HM(\chi )$	$({\mathfrak {y+z}})^2$	$(D_{{\mathfrak {y}}}+D_{{\mathfrak {z}}})^2({\mathbb {M}}(\chi ); {\mathfrak {y,z}})|_{{\mathfrak {y=z=1}}}$
$F(\chi )$	${\mathfrak {y^2+z^2}}$	$(D_{{\mathfrak {y}}}^2+D_{{\mathfrak {z}}}^2)({\mathbb {M}}(\chi ); {\mathfrak {y,z}})|_{{\mathfrak {y=z=1}}}$
$\sigma (\chi )$	$({\mathfrak {y-z}})^2$	$(D_{{\mathfrak {y}}}-D_{{\mathfrak {z}}})^2({\mathbb {M}}(\chi ); {\mathfrak {y,z}})|_{{\mathfrak {y=z=1}}}$
$SDD(\chi )$	$\frac{{\mathfrak {y^2+z^2}}}{{\mathfrak {yz}}}$	$(D_{{\mathfrak {y}}}S_{{\mathfrak {z}}}+D_{{\mathfrak {z}}}S_{{\mathfrak {y}}})({\mathbb {M}}(\chi ); {\mathfrak {y,z}})|_{{\mathfrak {y=z=1}}}$
$I(\chi )$	$\frac{{\mathfrak {yz}}}{{\mathfrak {y+z}}}$	$(S_{{\mathfrak {y}}}JD_{{\mathfrak {y}}}D_{{\mathfrak {z}}})({\mathbb {M}}(\chi ); {\mathfrak {y,z}})|_{{\mathfrak {y=1}}}$

The operators are required which relate the degree-based topological descriptors with the ${\mathbb {M}}$-polynomial,

$$\begin{aligned} D_{{\mathfrak {y}}}(\texttt{f}({\mathfrak {y,z}}))= & {} {{\mathfrak {y}}}\frac{\partial (\texttt{f}({\mathfrak {y,z}}))}{\partial {{\mathfrak {y}}}}, D_{{\mathfrak {z}}}(\texttt{f}({\mathfrak {y,z}}))={{\mathfrak {z}}}\frac{\partial (\texttt{f}({\mathfrak {y,z}}))}{\partial {{\mathfrak {z}}}}, S_{{\mathfrak {y}}}(\texttt{f}({\mathfrak {y,z}}))= \int _{0}^{{{\mathfrak {y}}}} \frac{\texttt{f}({\mathfrak {q,z}})}{{{\mathfrak {q}}}}d{{\mathfrak {q}}} \\ S_{{\mathfrak {z}}}(\texttt{f}({\mathfrak {y,z}}))= & {} \int _{0}^{{{\mathfrak {z}}}} \frac{\texttt{f}({\mathfrak {y,q}})}{{{\mathfrak {q}}}}d{{\mathfrak {q}}}, J(\texttt{f}({\mathfrak {y,z}}))= \texttt{f}({\mathfrak {y,y}}), Q_\kappa (\texttt{f}({\mathfrak {y,z}}))= {{\mathfrak {y}}}^\kappa \texttt{f}({\mathfrak {y,z}}); \kappa \ne 0. \end{aligned}$$

## Main results and discussion

### M-polynomial of boron $\alpha$-icosahedral nanosheet

#### Theorem 1

If $\chi = I_\alpha ({\mathfrak {s,p}})|{\mathfrak {s,p}}\ge 1$ represents a boron $\alpha$-icosahedral nanosheet, then ${\mathbb {M}}$-polynomial is

$$\begin{aligned} {\mathbb {M}}(I_\alpha ({\mathfrak {s,p}});{\mathfrak {y,z}})&=(4{\mathfrak {sp}}+14{{\mathfrak {s}}}+16{{\mathfrak {p}}}-4){\mathfrak {y^5z^5}} +(12{\mathfrak {sp}}-2{{\mathfrak {p}}}+2{{\mathfrak {s}}}-12) {\mathfrak {y^5z^6}}\\&\quad +(18{\mathfrak {sp}}-17{{\mathfrak {p}}}-19{{\mathfrak {s}}}+18) {\mathfrak {y^6z^6}} \end{aligned}$$

**Figure 3:**
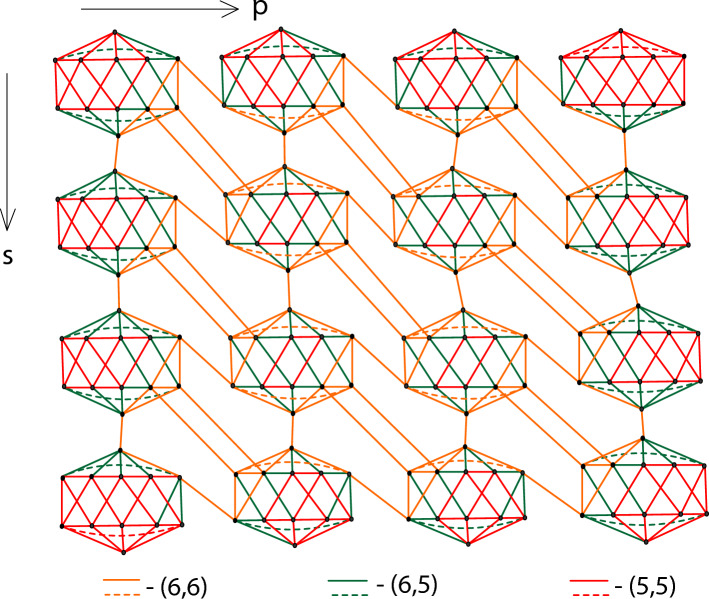
Edge partition of boron $\alpha$-icosahedral nanosheet, $I_\alpha ({\mathfrak {4,4}})$.

#### Proof

The boron $\alpha$-icosahedral nanosheet, $I_\alpha ({\mathfrak {s,p}})|{\mathfrak {s,p}}\ge 1$ contains $12{\mathfrak {sp}}$ vertices and $34{\mathfrak {sp}}-3{{\mathfrak {s}}}-3{{\mathfrak {p}}}+2$ edges. icosahedral nanosheets are categorized into edge sets based on the degree of the vertex, ${{\mathfrak {D}}}=\{(5,5), (5,6), (6,6)\}$. The edge partition of $I_\alpha ({\mathfrak {s,p}})$ based on the vertex degree is depicted in Fig. 3. The edge sets, ${\mathcal {E}}(I_\alpha ({\mathfrak {s,p}}))$ is classified into three types and the parameter value, $\texttt{d}_{{\mathfrak {kh}}}$ is characterized by the following value,

$$\begin{aligned} \texttt{d}_{55}&= |\{\mu \eta \in {\mathcal {E}}(I_\alpha ({\mathfrak {s,p}})|\texttt{d}_\mu = 5 \text { and } \texttt{d}_\eta = 5\}|= 4{\mathfrak {sp}}+14{{\mathfrak {s}}}+16{{\mathfrak {p}}}-4\\ \texttt{d}_{56}&= |\{\mu \eta \in {\mathcal {E}}(I_\alpha ({\mathfrak {s,p}})|\texttt{d}_\mu = 5 \text { and } \texttt{d}_\eta = 6\}|= 12{\mathfrak {sp}}+2{{\mathfrak {s}}}-2{{\mathfrak {p}}}-12\\ \texttt{d}_{66}&= |\{\mu \eta \in {\mathcal {E}}(I_\alpha ({\mathfrak {s,p}})|\texttt{d}_\mu = 6 \text { and } \texttt{d}_\eta = 6\}|= 18{\mathfrak {sp}}-19{{\mathfrak {s}}}-17{{\mathfrak {p}}}+18 \end{aligned}$$

By the Definition (1), ${\mathbb {M}}$-polynomial of boron $\alpha$-icosahedral nanosheet, $I_\alpha ({\mathfrak {s,p}})|{\mathfrak {s,p}}\ge 1$ is defined as

$$\begin{aligned} {\mathbb {M}}(I_\alpha ({\mathfrak {s,p}});{\mathfrak {y,z}})= \sum _{{{\mathfrak {k}}}\le {{\mathfrak {h}}}}\texttt{m}_{{\mathfrak {kh}}}(I_\alpha ({\mathfrak {s,p}})){{\mathfrak {y}}}^{{{\mathfrak {k}}}}{{\mathfrak {z}}}^{{{\mathfrak {h}}}} \end{aligned}$$

**Figure 4:**
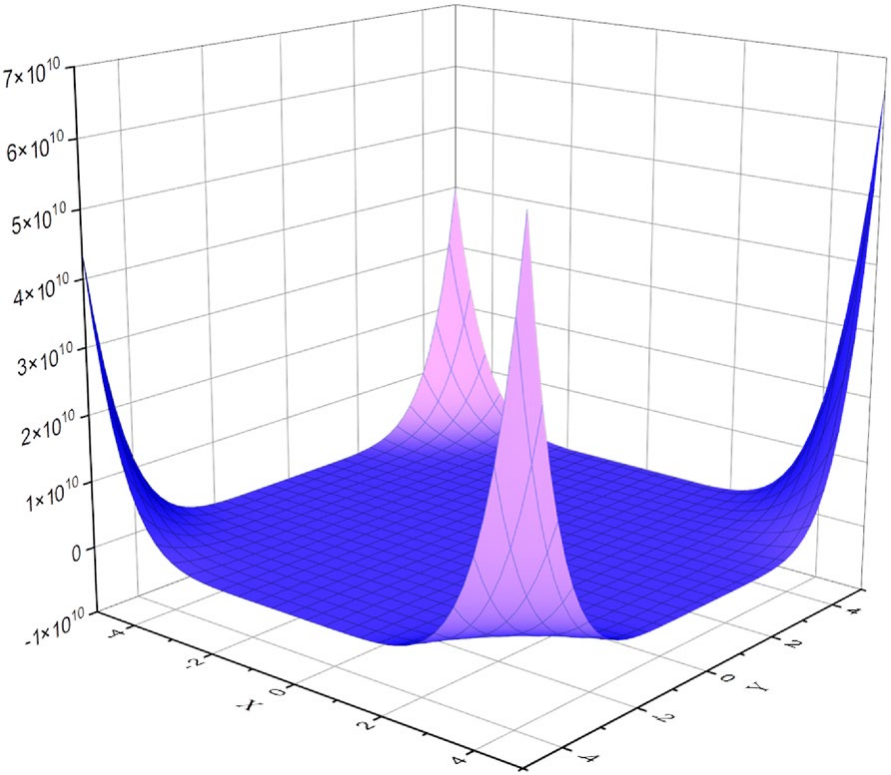
${\mathbb {M}}$-polynomial of boron $\alpha$-icosahedral nanosheet, $I_\alpha ({\mathfrak {4,5}})$.

Figure 4 shows the graphical illustration of the ${\mathbb {M}}$-polynomial function of $I_\alpha ({\mathfrak {s,p}})|{{\mathfrak {s}}}=4 \text { and }{{\mathfrak {p}}}=5$. Thus, ${\mathbb {M}}(I_\alpha ({\mathfrak {s,p}});{\mathfrak {y,z}})$ can be formulated as,

$$\begin{aligned} {\mathbb {M}}(I_\alpha ({\mathfrak {s,p}});{\mathfrak {y,z}})&= \sum _{{{\mathfrak {5}}}\le {{\mathfrak {5}}}}\texttt{m}_{{\mathfrak {55}}}(I_\alpha ({\mathfrak {s,p}})){{\mathfrak {y}}}^{{{\mathfrak {5}}}}{{\mathfrak {z}}}^{{{\mathfrak {5}}}}+ \sum _{{{\mathfrak {5}}}\le {{\mathfrak {6}}}}\texttt{m}_{{\mathfrak {56}}}(I_\alpha ({\mathfrak {s,p}})){{\mathfrak {y}}}^{{{\mathfrak {5}}}}{{\mathfrak {z}}}^{{{\mathfrak {6}}}}+ \sum _{{{\mathfrak {6}}}\le {{\mathfrak {6}}}}\texttt{m}_{{\mathfrak {66}}}(I_\alpha ({\mathfrak {s,p}})){{\mathfrak {y}}}^{{{\mathfrak {6}}}}{{\mathfrak {z}}}^{{{\mathfrak {6}}}}\\&= \texttt{d}_{55}{{\mathfrak {y}}}^{{{\mathfrak {5}}}}{{\mathfrak {z}}}^{{{\mathfrak {5}}}}+ \texttt{d}_{56}{{\mathfrak {y}}}^{{{\mathfrak {5}}}}{{\mathfrak {z}}}^{{{\mathfrak {6}}}}+ \texttt{d}_{66}{{\mathfrak {y}}}^{{{\mathfrak {6}}}}{{\mathfrak {z}}}^{{{\mathfrak {6}}}}\\&=(4{\mathfrak {sp}}+14{{\mathfrak {s}}}+16{{\mathfrak {p}}}-4){\mathfrak {y^5z^5}} +(12{\mathfrak {sp}}-2{{\mathfrak {p}}}+2{{\mathfrak {s}}}-12) {\mathfrak {y^5z^6}}\\&\qquad +(18{\mathfrak {sp}}-17{{\mathfrak {p}}}-19{{\mathfrak {s}}}+18) {\mathfrak {y^6z^6}} \end{aligned}$$

### Results for boron $\alpha$-icosahedral nanosheet

**Figure 5:**
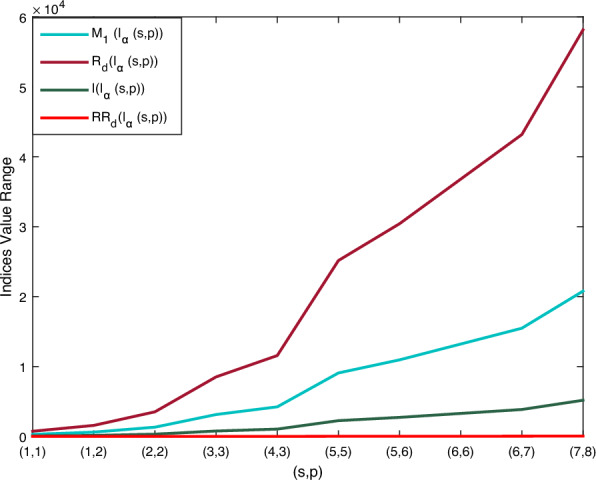
Visualization of $$M_1(I_\alpha ({\mathfrak {s,p}})),$$ $$R_{{\mathfrak {d}}}(I_\alpha ({\mathfrak {s,p}})),$$ $$I(I_\alpha ({\mathfrak {s,p}})),$$ $$RR_{{\mathfrak {d}}}(I_\alpha ({\mathfrak {s,p}}))$$.

**Figure 6:**
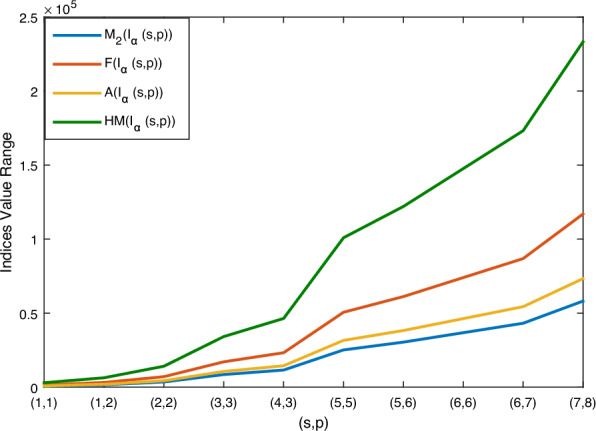
Graphical representation of $M_2(I_\alpha ({\mathfrak {s,p}}))$ $F(I_\alpha ({\mathfrak {s,p}}))$ $A(I_\alpha ({\mathfrak {s,p}}))$ $HM(I_\alpha ({\mathfrak {s,p}}))$.

**Figure 7:**
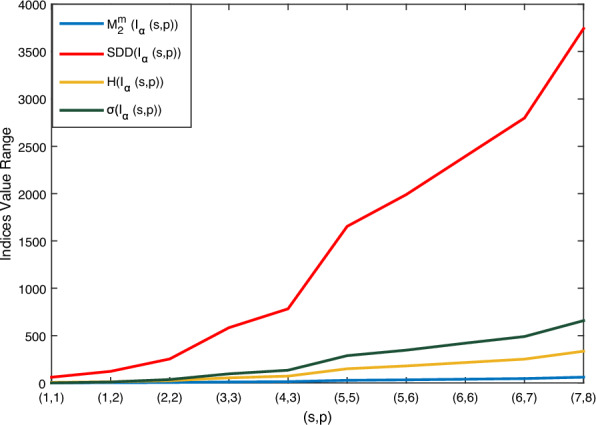
Graphical illustration of $M_2^m(I_\alpha ({\mathfrak {s,p}})),$ $SDD(I_\alpha ({\mathfrak {s,p}})),$ $H(I_\alpha ({\mathfrak {s,p}})),$ $\sigma (I_\alpha ({\mathfrak {s,p}}))$.

Using Theorem 1 and ${\mathbb {M}}$-polynomial formula in Table 1, vertex degree-based topological indices such as $M_1, M_2, M_2^m, A, R_{{\mathfrak {d}}}, RR_{{\mathfrak {d}}}, H, HM, F, \sigma , SDD \text { and } I$ of boron $\alpha$-icosahedral nanosheet, $I_\alpha ({\mathfrak {s,p}})|{\mathfrak {s,p}}\ge 1$ are computed. Here, $\texttt{f}({\mathfrak {y,z}})= {\mathbb {M}}(I_\alpha ({\mathfrak {s,p}});{\mathfrak {y,z}})=(4{\mathfrak {sp}}+14{{\mathfrak {s}}}+16{{\mathfrak {p}}}-4){\mathfrak {y^5z^5}} +(12{\mathfrak {sp}}-2{{\mathfrak {p}}}+2{{\mathfrak {s}}}-12) {\mathfrak {y^5z^6}}+(18{\mathfrak {sp}}-17{{\mathfrak {p}}}-19{{\mathfrak {s}}}+18) {\mathfrak {y^6z^6}}$. The numerical value of the derived analytical expression is compared with each index is depicted in Tables 2, 3, 4 and 5. And the graphical comparison is illustrated in Figs. 5, 6 and 7.

#### Theorem 2

Let $I_\alpha ({\mathfrak {s,p}})|{\mathfrak {s,p}}\ge 1$ be a boron $\alpha$-icosahedral nanosheet then

1. $$M_1(I_\alpha ({\mathfrak {s,p}})) =388{{\mathfrak {s}}}{{\mathfrak {p}}}-66{{\mathfrak {p}}}-66{{\mathfrak {s}}}+44$$
2. $$R_{{\mathfrak {d}}}(I_\alpha ({\mathfrak {s,p}})) = 1108{\mathfrak {sp}}-272{{\mathfrak {p}}}-274{{\mathfrak {s}}}+188$$
3. $$I(I_\alpha ({\mathfrak {s,p}})= \displaystyle \frac{1064{\mathfrak {sp}}}{11}-\frac{181{{\mathfrak {p}}}}{11}-\frac{182{{\mathfrak {s}}}}{11}+\frac{124}{11}$$
4. $$RR_{{\mathfrak {d}}}(I_\alpha ({\mathfrak {s,p}}) = \displaystyle \frac{89{{\mathfrak {s}}}}{900}+\frac{91{{\mathfrak {p}}}}{900}+\frac{53{\mathfrak {sp}}}{50}-\frac{3}{50}$$

#### Proof

1. $$M_1(I_\alpha ({\mathfrak {s,p}})) = (D_{{\mathfrak {y}}}+D_{{\mathfrak {z}}})({\mathbb {M}}(I_\alpha ({\mathfrak {s,p}}));{\mathfrak {y,z}})|_{{\mathfrak {y=z=1}}}$$
  $$\begin{aligned} (D_{{\mathfrak {y}}}+D_{{\mathfrak {z}}})(\texttt{f}({\mathfrak {y,z}}))&= 2{{\mathfrak {y}}}^5{{\mathfrak {z}}}^5(70{{\mathfrak {s}}}+80{{\mathfrak {p}}}-66{{\mathfrak {z}}}+20{{\mathfrak {s}}}{{\mathfrak {p}}}+11{{\mathfrak {s}}}{{\mathfrak {z}}}-11{{\mathfrak {p}}}{{\mathfrak {z}}}+108{{\mathfrak {y}}}{{\mathfrak {z}}}+66{{\mathfrak {s}}}{{\mathfrak {p}}}{{\mathfrak {z}}}\\ {}&\qquad -114{{\mathfrak {s}}}{{\mathfrak {y}}}{{\mathfrak {z}}}-102{{\mathfrak {p}}}{{\mathfrak {y}}}{{\mathfrak {z}}}+108{{\mathfrak {s}}}{{\mathfrak {p}}}{{\mathfrak {y}}}{{\mathfrak {z}}}-20)\big |_{{\mathfrak {y=z=1}}}\\&= 388{{\mathfrak {s}}}{{\mathfrak {p}}}-66{{\mathfrak {p}}}-66{{\mathfrak {s}}}+44 \end{aligned}$$
2. $$R_{{\mathfrak {d}}}(I_\alpha ({\mathfrak {s,p}})) = (D_{{\mathfrak {y}}}^{{\mathfrak {d}}}+D_{{\mathfrak {z}}}^{{\mathfrak {d}}})({\mathbb {M}}(I_\alpha ({\mathfrak {s,p}})); {\mathfrak {y,z}})|_{{\mathfrak {y=z=1}}}$$
  $$\begin{aligned} (D_{{\mathfrak {y}}}^{{\mathfrak {d}}}+D_{{\mathfrak {z}}}^{{\mathfrak {d}}})(\texttt{f}({\mathfrak {y,z}}))&= 5^{2{{\mathfrak {d}}}}(14{{\mathfrak {s}}}+16{{\mathfrak {p}}}+4{\mathfrak {sp}}-4) -6^{2{{\mathfrak {d}}}}(19{{\mathfrak {s}}}+17{{\mathfrak {p}}}-18{\mathfrak {sp}}-18)\\&\quad +5^{{\mathfrak {d}}}6^{{\mathfrak {d}}}(2{{\mathfrak {s}}}-2{{\mathfrak {p}}}+12{\mathfrak {sp}}-12)\Big |_{{\mathfrak {y=z=1}}; {{\mathfrak {d}}}=1}\\&= 1108{\mathfrak {sp}}-272{{\mathfrak {p}}}-274{{\mathfrak {s}}}+188 \end{aligned}$$
3. $$I(I_\alpha ({\mathfrak {s,p}}))= (S_{{\mathfrak {y}}}JD_{{\mathfrak {y}}}D_{{\mathfrak {z}}})({\mathbb {M}}(I_\alpha ({\mathfrak {s,p}})); {\mathfrak {y,z}})$$
  $$\begin{aligned} (S_{{\mathfrak {y}}}JD_{{\mathfrak {y}}}D_{{\mathfrak {z}}})(\texttt{f}({\mathfrak {y,z}}))&= {{\mathfrak {y}}}^{12}(54{\mathfrak {sp}}-51{{\mathfrak {p}}}-57{{\mathfrak {s}}}+54) +{{\mathfrak {y}}}^{11}\bigg (\frac{60{{\mathfrak {s}}}}{11}-\frac{60{{\mathfrak {p}}}}{11}\\&\quad +\frac{360{\mathfrak {sp}}}{11}-\frac{360}{11}\bigg )+{{\mathfrak {y}}}^{10}(10{\mathfrak {sp}}+35{{\mathfrak {s}}}+40{{\mathfrak {p}}}-10)\Big |_{{\mathfrak {y=1}}}\\&= \frac{1064{\mathfrak {sp}}}{11}-\frac{181{{\mathfrak {p}}}}{11}-\frac{182{{\mathfrak {s}}}}{11}+\frac{124}{11} \end{aligned}$$
4. $$RR_{{\mathfrak {d}}}(I_\alpha ({\mathfrak {s,p}}) = S_{{\mathfrak {y}}}^{{\mathfrak {d}}} S_{{\mathfrak {z}}}^{{\mathfrak {d}}}(D_{{\mathfrak {y}}}+D_{{\mathfrak {z}}})({\mathbb {M}}(I_\alpha ({\mathfrak {s,p}})); {\mathfrak {y,z}})$$
  $$\begin{aligned} S_{{\mathfrak {y}}}^{{\mathfrak {d}}} S_{{\mathfrak {z}}}^{{\mathfrak {d}}}(\texttt{f}({\mathfrak {y,z}}))&= \frac{1}{5^{2{{\mathfrak {d}}}}}(14{{\mathfrak {s}}}+16{{\mathfrak {p}}}+4{\mathfrak {sp}}-4)+\frac{1}{30^{{\mathfrak {d}}}}(2{{\mathfrak {s}}}-2{{\mathfrak {p}}}+12{\mathfrak {sp}}-12)\\ {}&\qquad \frac{-1}{6^{2{{\mathfrak {d}}}}}(19{{\mathfrak {s}}}+17{{\mathfrak {p}}}-18{\mathfrak {sp}}-18)\Big |_{{\mathfrak {y=z=1}}; {{\mathfrak {d}}}=1} \\&= \frac{89{{\mathfrak {s}}}}{900}+\frac{91{{\mathfrak {p}}}}{900}+\frac{53{\mathfrak {sp}}}{50}-\frac{3}{50} \end{aligned}$$

**Table 2:** Numerical value of $M_1(I_\alpha ({\mathfrak {s,p}}))$ $R_{{\mathfrak {d}}}(I_\alpha ({\mathfrak {s,p}}))$ $I(I_\alpha ({\mathfrak {s,p}}))$ $RR_{{\mathfrak {d}}}(I_\alpha ({\mathfrak {s,p}}))$.

$({\mathfrak {s,p}})$	$M_1(I_\alpha ({\mathfrak {s,p}}))$	$R_{{\mathfrak {d}}}(I_\alpha ({\mathfrak {s,p}}))$	$I(I_\alpha ({\mathfrak {s,p}}))$	$RR_{{\mathfrak {d}}}(I_\alpha ({\mathfrak {s,p}}))$
(1, 1)	300	750	75	1.2
(1, 2)	622	1586	155.2727	2.3611
(2, 2)	1332	3528	332.1818	4.58
(3, 3)	3140	8522	782.8182	10.08
(4, 3)	4238	11572	1056.4545	13.3589
(5, 5)	9084	25158	2264.4545	27.44
(5, 6)	10958	30426	2731.6364	32.8411
(6, 6)	13220	36800	3295.4545	39.3
(6, 7)	15482	43176	3859.3636	45.7611
(7, 8)	20782	58142	5180.5454	60.8011

#### Theorem 3

Let $I_\alpha ({\mathfrak {s,p}})|{\mathfrak {s,p}}\ge 1$ be a boron $\alpha$-icosahedral nanosheet then

1. $$M_2(I_\alpha ({\mathfrak {s,p}}))= 1108{{\mathfrak {s}}}{{\mathfrak {p}}}-272{{\mathfrak {p}}}-274{{\mathfrak {s}}}+188$$
2. $$F(I_\alpha ({\mathfrak {s,p}}))= 2228{\mathfrak {sp}}-546{{\mathfrak {p}}}-546{{\mathfrak {s}}}+364$$
3. $$A(I_\alpha ({\mathfrak {s,p}})= \displaystyle \frac{202510477{\mathfrak {sp}}}{144000}- \frac{40926041{{\mathfrak {p}}}}{108000} - \frac{332764271{{\mathfrak {s}}}}{864000}+ \frac{39354227}{144000}$$
4. $$HM(I_\alpha ({\mathfrak {s,p}}))= 4444{\mathfrak {sp}}-1090{{\mathfrak {p}}}-1094{{\mathfrak {s}}}+740$$

#### Proof

1. $$M_2(I_\alpha ({\mathfrak {s,p}})) = D_{{\mathfrak {y}}}D_{{\mathfrak {z}}}({\mathbb {M}}(I_\alpha ({\mathfrak {s,p}}));{\mathfrak {y,z}})$$
  $$\begin{aligned} D_{{\mathfrak {y}}}D_{{\mathfrak {z}}}(\texttt{f}({\mathfrak {y,z}}))&={{\mathfrak {y}}}{{\mathfrak {z}}} \big (30{{\mathfrak {y}}}^4{{\mathfrak {z}}}^5(2{{\mathfrak {s}}}-2{{\mathfrak {p}}}+12{{\mathfrak {s}}}{{\mathfrak {p}}}-12) +25{{\mathfrak {y}}}^4{{\mathfrak {z}}}^4(14{{\mathfrak {s}}}+16{{\mathfrak {p}}}+4{{\mathfrak {s}}}{{\mathfrak {p}}}-4)\\&\quad -36{{\mathfrak {y}}}^5{{\mathfrak {z}}}^5(19{{\mathfrak {s}}}+17{{\mathfrak {p}}}-18{{\mathfrak {s}}}{{\mathfrak {p}}}-18)\big )\big |_{{\mathfrak {y=z=1}}}\\&= 1108{{\mathfrak {s}}}{{\mathfrak {p}}}-272{{\mathfrak {p}}}-274{{\mathfrak {s}}}+188 \end{aligned}$$
2. $$F(I_\alpha ({\mathfrak {s,p}}))= (D_{{\mathfrak {y}}}^2+D_{{\mathfrak {z}}}^2)({\mathbb {M}}(I_\alpha ({\mathfrak {s,p}})); {\mathfrak {y,z}})$$
  $$\begin{aligned} (D_{{\mathfrak {y}}}^2+D_{{\mathfrak {z}}}^2)(\texttt{f}({\mathfrak {y,z}}))&= 2{{\mathfrak {y}}}^5{{\mathfrak {z}}}^5(350{{\mathfrak {s}}}+400{{\mathfrak {p}}}-366{{\mathfrak {z}}}+100{{\mathfrak {s}}}{{\mathfrak {p}}}+61{{\mathfrak {s}}}{{\mathfrak {z}}}-61{{\mathfrak {p}}}{{\mathfrak {z}}}+648{\mathfrak {yz}}\\ {}&\qquad +366{\mathfrak {spz}}-684{\mathfrak {syz}}-612{\mathfrak {pyz}}+648{\mathfrak {psyz}}-100)\Big |_{{\mathfrak {y=z=1}}} \\&= 2228{\mathfrak {sp}}-546{{\mathfrak {p}}}-546{{\mathfrak {s}}}+364 \end{aligned}$$
3. $$A(I_\alpha ({\mathfrak {s,p}}))= (S_{{\mathfrak {y}}}^3Q_{-2}D_{{\mathfrak {y}}}^3D_{{\mathfrak {z}}}^3)({\mathbb {M}}(I_\alpha ({\mathfrak {s,p}})); {\mathfrak {y,z}})$$
  $$\begin{aligned} (S_{{\mathfrak {y}}}^3Q_{-2}D_{{\mathfrak {y}}}^3D_{{\mathfrak {z}}}^3)(\texttt{f}({\mathfrak {y,z}}))&= {{\mathfrak {y}}}^{10}\bigg (\frac{104976{{\mathfrak {s}}}{{\mathfrak {p}}}}{125}-\frac{99144{{\mathfrak {p}}}}{125}-\frac{110808{{\mathfrak {s}}}}{125}+\frac{104976}{125}\bigg )\\ {}&\qquad +{{\mathfrak {y}}}^9\bigg (\frac{2000{{\mathfrak {s}}}}{27}-\frac{2000{{\mathfrak {p}}}}{27}+\frac{4000{{\mathfrak {s}}}{{\mathfrak {p}}}}{9}-\frac{4000}{9}\bigg )\\ {}&\qquad +{{\mathfrak {y}}}^8\bigg (\frac{109375{{\mathfrak {s}}}}{256}+\frac{15625{{\mathfrak {p}}}}{32}+\frac{15625{{\mathfrak {s}}}{{\mathfrak {p}}}}{128}-\frac{15625}{128}\bigg )\Big |_{{\mathfrak {y=1}}}\\&= \frac{202510477{\mathfrak {sp}}}{144000}- \frac{40926041{{\mathfrak {p}}}}{108000} - \frac{332764271{{\mathfrak {s}}}}{864000}+\frac{39354227}{144000} \end{aligned}$$
4. $$HM(I_\alpha ({\mathfrak {s,p}}))= (D_{{\mathfrak {y}}}+D_{{\mathfrak {z}}})^2({\mathbb {M}}(I_\alpha ({\mathfrak {s,p}})); {\mathfrak {y,z}})$$
  $$\begin{aligned} (D_{{\mathfrak {y}}}+D_{{\mathfrak {z}}})^2(\texttt{f}({\mathfrak {y,z}}))&= 2{{\mathfrak {y}}}^5{{\mathfrak {z}}}^5(700{{\mathfrak {s}}}+800{{\mathfrak {p}}}-726{{\mathfrak {z}}}+200{{\mathfrak {s}}}{{\mathfrak {p}}}+121{{\mathfrak {s}}}{{\mathfrak {z}}}-121{{\mathfrak {p}}}{{\mathfrak {z}}}+1296{\mathfrak {yz}}\\ {}&\qquad +726{\mathfrak {spz}}-1368{\mathfrak {syz}}-1224{\mathfrak {pyz}}+1296{\mathfrak {spyz}}-200)\Big |_{{\mathfrak {y=z=1}}}\\&= 4444{\mathfrak {sp}}-1090{{\mathfrak {p}}}-1094{{\mathfrak {s}}}+740 \end{aligned}$$

**Table 3:** Numerical value of $M_2(I_\alpha ({\mathfrak {s,p}}))$ $F(I_\alpha ({\mathfrak {s,p}}))$ $A(I_\alpha ({\mathfrak {s,p}}))$ $HM(I_\alpha ({\mathfrak {s,p}}))$.

$({\mathfrak {s,p}})$	$M_2(I_\alpha ({\mathfrak {s,p}}))$	$F(I_\alpha ({\mathfrak {s,p}}))$	$A(I_\alpha ({\mathfrak {s,p}}))$	$HM(I_\alpha ({\mathfrak {s,p}}))$
(1, 1)	750	1500	915.5273	3000
(1, 2)	1586	3182	1942.9053	6354
(2, 2)	3528	7092	4370.407	14148
(3, 3)	8522	17140	10637.9321	34184
(4, 3)	11572	23278	14471.7565	46422
(5, 5)	25158	50604	31610.9189	100920
(5, 6)	30426	61198	38263.5878	122050
(6, 6)	36800	74020	46316.3806	147620
(6, 7)	43176	86842	54375.3723	173194
(7, 8)	58142	116942	73299.8022	233226

#### Theorem 4

Let $I_\alpha ({\mathfrak {s,p}})|{\mathfrak {s,p}}\ge 1$ be a boron $\alpha$-icosahedral nanosheet then

1. $$M_2^m(I_\alpha ({\mathfrak {s,p}}))= \displaystyle \frac{89{{\mathfrak {s}}}}{900}+\frac{91{{\mathfrak {p}}}}{900}+\frac{53{\mathfrak {sp}}}{50}-\frac{3}{50}$$
2. $$SDD(I_\alpha ({\mathfrak {s,p}}))=\displaystyle \frac{342{\mathfrak {sp}}}{5}-\frac{91{{\mathfrak {p}}}}{15}-\frac{89{{\mathfrak {s}}}}{15}+\frac{18}{5}$$
3. $$H(I_\alpha ({\mathfrak {s,p}})) = \displaystyle \frac{{{\mathfrak {p}}}}{330}-\frac{{{\mathfrak {s}}}}{330}+\frac{329{\mathfrak {sp}}}{55}+\frac{1}{55}$$
4. $$\sigma (I_\alpha ({\mathfrak {s,p}}))= 2{{\mathfrak {s}}}-2{{\mathfrak {p}}}+12{\mathfrak {sp}}-12$$

#### Proof

1. $$M_2^m(I_\alpha ({\mathfrak {s,p}})) = S_{{\mathfrak {y}}}S_{{\mathfrak {z}}}({\mathbb {M}}(I_\alpha ({\mathfrak {s,p}}));{\mathfrak {y,z}})$$
  $$\begin{aligned} S_{{\mathfrak {y}}}S_{{\mathfrak {z}}}(\texttt{f}({\mathfrak {y,z}}))&= \frac{{{\mathfrak {y}}}^5{{\mathfrak {z}}}^6(2{{\mathfrak {s}}} -2{{\mathfrak {p}}}+12{{\mathfrak {s}}}{{\mathfrak {p}}}-12)}{30}+\frac{{{\mathfrak {y}}}^5{{\mathfrak {z}}}^5(14{{\mathfrak {s}}}+ 16{{\mathfrak {p}}}+4{{\mathfrak {s}}}{{\mathfrak {p}}}-4)}{25}\\&\quad -\frac{{{\mathfrak {y}}}^6{{\mathfrak {z}}}^6(19{{\mathfrak {s}}}+17{{\mathfrak {p}}}-18{{\mathfrak {s}}}{{\mathfrak {p}}}-18)}{36} \Big |_{{\mathfrak {y=z=1}}}\\&= \frac{89{{\mathfrak {s}}}}{900}+\frac{91{{\mathfrak {p}}}}{900}+\frac{53{\mathfrak {sp}}}{50}-\frac{3}{50} \end{aligned}$$
2. $$SDD(I_\alpha ({\mathfrak {s,p}}))= (D_{{\mathfrak {y}}}S_{{\mathfrak {z}}}+D_{{\mathfrak {z}}}S_{{\mathfrak {y}}})({\mathbb {M}}(I_\alpha ({\mathfrak {s,p}})); {\mathfrak {y,z}})$$
  $$\begin{aligned} (D_{{\mathfrak {y}}}S_{{\mathfrak {z}}}+D_{{\mathfrak {z}}}S_{{\mathfrak {y}}})(\texttt{f}({\mathfrak {y,z}}))&= \frac{{\mathfrak {y^5z^5}}}{15}\big (420{{\mathfrak {s}}}+480{{\mathfrak {p}}}-366{{\mathfrak {z}}}+120{\mathfrak {sp}}+61{\mathfrak {sz}}-61{\mathfrak {pz}}+540{\mathfrak {yz}}\\ {}&\qquad +366{\mathfrak {spz}}-570{\mathfrak {syz}}-510{\mathfrak {pyz}}+540{\mathfrak {spyz}}-120\big ) \Big |_{{\mathfrak {y=z=1}}}\\&= \frac{342{\mathfrak {sp}}}{5}-\frac{91{{\mathfrak {p}}}}{15}-\frac{89{{\mathfrak {s}}}}{15}+\frac{18}{5} \end{aligned}$$
3. $$H(I_\alpha ({\mathfrak {s,p}})) = 2S_{{\mathfrak {y}}}J({\mathbb {M}}(I_\alpha ({\mathfrak {s,p}})); {\mathfrak {y,z}})$$
  $$\begin{aligned} 2S_{{\mathfrak {y}}}J(\texttt{f}({\mathfrak {y,z}}))&= 2{{\mathfrak {y}}}^{10}\bigg (\frac{7{{\mathfrak {s}}}}{5} +\frac{8{{\mathfrak {p}}}}{5}+\frac{2{\mathfrak {sp}}}{5}-\frac{2}{5}\bigg )-2{{\mathfrak {y}}}^{12} \bigg (\frac{19{{\mathfrak {s}}}}{12}+\frac{17{{\mathfrak {p}}}}{12}-\frac{3{\mathfrak {sp}}}{2}-\frac{3}{2}\bigg )\\&\quad +2{{\mathfrak {y}}}^{11}\bigg (\frac{2{{\mathfrak {s}}}}{11}-\frac{2{{\mathfrak {p}}}}{11}+\frac{12{\mathfrak {sp}}}{11}-\frac{12}{11}\bigg ) \Big |_{{\mathfrak {y=1}}}\\&= \frac{{{\mathfrak {p}}}}{330}-\frac{{{\mathfrak {s}}}}{330}+\frac{329{\mathfrak {sp}}}{55}+\frac{1}{55} \end{aligned}$$
4. $$\sigma (I_\alpha ({\mathfrak {s,p}}))= (D_{{\mathfrak {y}}}-D_{{\mathfrak {z}}})^2({\mathbb {M}}(I_\alpha ({\mathfrak {s,p}})); {\mathfrak {y,z}})$$
  $$\begin{aligned} (D_{{\mathfrak {y}}}-D_{{\mathfrak {z}}})^2(\texttt{f}({\mathfrak {y,z}}))&= 2{\mathfrak {y^5z^6}}({{\mathfrak {s}}}-{{\mathfrak {p}}}+6{\mathfrak {sp}}-6)\Big |_{{\mathfrak {y=z=1}}}\\&= 2{{\mathfrak {s}}}-2{{\mathfrak {p}}}+12{\mathfrak {sp}}-12 \end{aligned}$$

**Table 4:** Numerical value of $M_2^m(I_\alpha ({\mathfrak {s,p}})),$ $SDD(I_\alpha ({\mathfrak {s,p}})),$ $H(I_\alpha ({\mathfrak {s,p}})),$ $\sigma (I_\alpha ({\mathfrak {s,p}}))$.

$({\mathfrak {s,p}})$	$M_2^m(I_\alpha ({\mathfrak {s,p}}))$	$SDD(I_\alpha ({\mathfrak {s,p}}))$	$H(I_\alpha ({\mathfrak {s,p}}))$	$\sigma (I_\alpha ({\mathfrak {s,p}}))$
(1, 1)	1.2	60	6	0
(1, 2)	2.3611	122.3333	11.9848	10
(2, 2)	4.58	253.2	23.9455	36
(3, 3)	10.08	583.2	53.8545	96
(4, 3)	13.3589	782.4667	71.797	134
(5, 5)	27.44	1653.6	149.5636	288
(5, 6)	32.8411	1989.5333	179.4758	346
(6, 6)	39.3	2394	215.3637	420
(6, 7)	45.7611	2798.3333	251.2576	490
(7, 8)	60.8011	3743.9333	335.003	658

## Properties prediction of boron crystal sheet

By emphasizing topological descriptors’ importance in QSAR/QSPR research and illustrating their predictive and assessment factors for boron sheets, a key focus of this study is described in this section. Using regression analysis, an equation has been formulated to relate the topological descriptors and the significant properties of boron sheets. With the aid of these formulations, one may further predict the characteristics of boron sheets, independent of their dimensions.

### Significance of molecular descriptors

In quantitative structure-activity relationship (QSAR) and quantitative structure-property relationship (QSPR), the topological descriptors, ’describes’ the molecular structure’s properties or activities in mathematical terminologies. QSAR/QSPR mathematically correlates the physicochemical properties or biological activity of chemical compounds with molecular descriptors. The base for this idea, QSAR/QSPR modelling is many chemical compounds have been implicitly equated with the overall risks which cause acute effects on human health. Some of the pesticide compounds are highly toxic and few may cause cancer.

**Figure 8:**
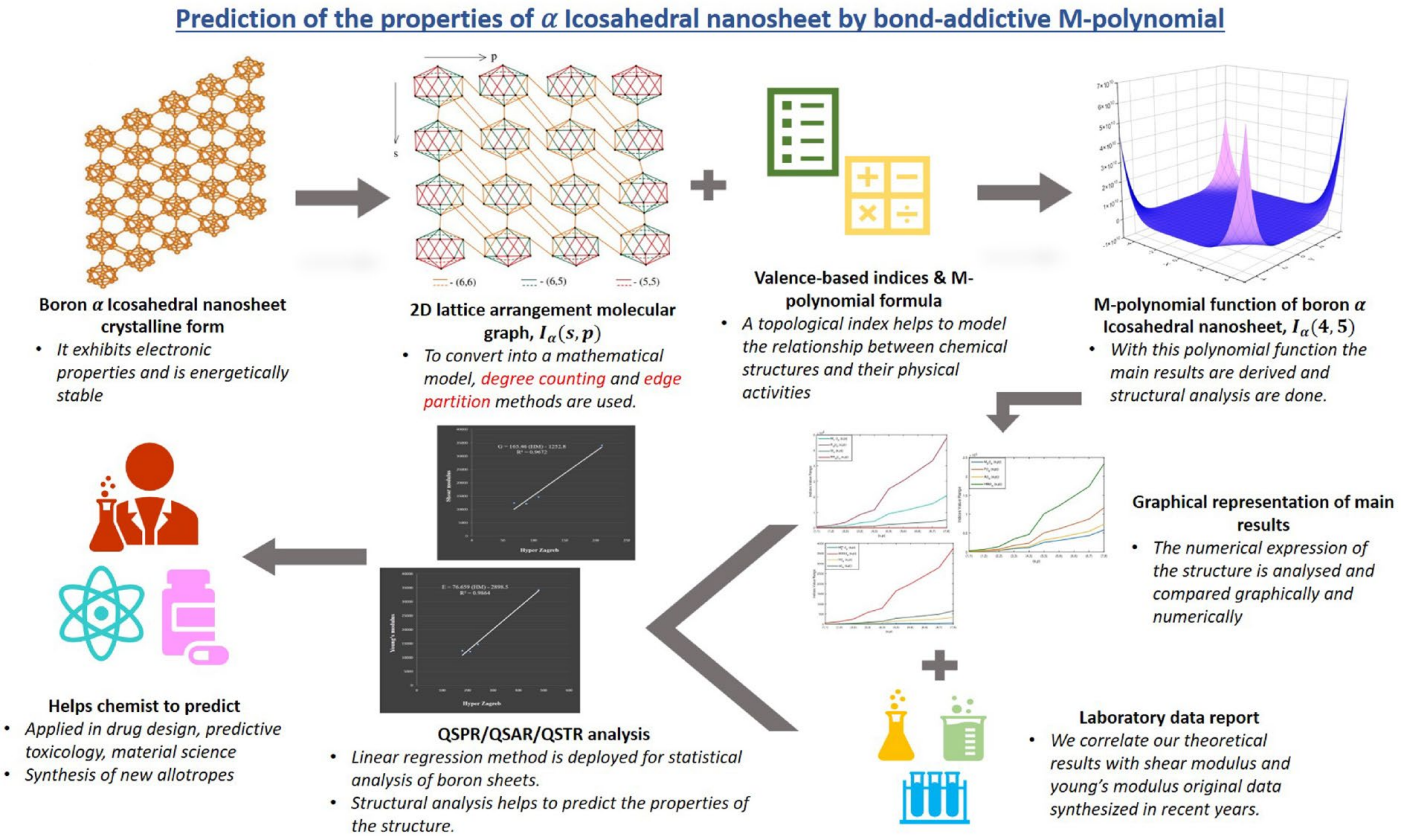
Graphical flowchart indicating the topological descriptors significance.

The toxicological testing of an active ingredient is usually limited. To estimate and rank the potentially hazardous chemicals, it is essential to develop an accurate and simple method^33^. Thus, it is a critical need to analyze and understand the structural properties of molecular compounds. Linear regression, multiple linear regression, logistic regression^34^, efficient linear method^35^, principal component analysis^36^, partial least square regression^37^, decision tree^38^ and random forest^39^ are the modelling techniques or methods that are used to analyze or predict the molecular compounds. In our study, linear regression method is deployed for statistical analysis of boron $\alpha$-icosahedral nanosheet. The graphica flowchart insisting on the topological descriptors and their potential uses is exhibited in Fig. 8.

### Variant of boron sheets and its descriptors

**Figure 9:**
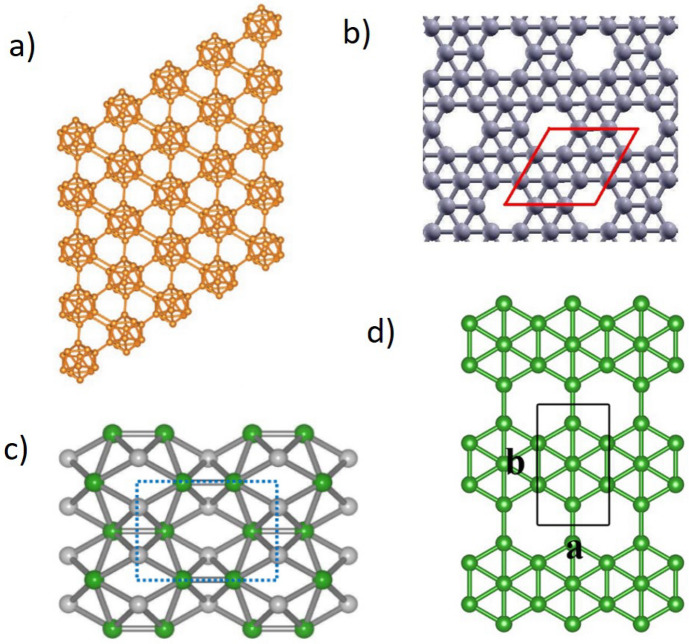
Boron nanosheet and its allotropes; (**a**) $\alpha$-icosahedral, $\alpha$-$B_{12}$ (**b**) $\alpha$ borophene (**c**) $8-Pmmn$ borophene (**d**) $\beta _{12}-$borphene.

Boron has recently received a lot of attention due to its diverse chemical properties and similarities to carbon. Due to the large number of allotropes and complex bonding nature of boron, many are interested to study its crystal structures and stability^40^. Icosahedra exhibit electrical and structural stability as well as interesting chemical bonding characteristics. A few of the two-dimensional boron sheets such as boron $\alpha$-icosahedral nanosheet, $\alpha$-$B_{12}$^2^, $\alpha$ borophene nanosheet^41^, $8-Pmmn$ borophene nanosheet^42^, $\beta _{12}$-borphene nanosheet^43^ were analyzed through regression analysis. The above-mentioned boron sheet is illustrated in Fig. 9. The degree-vertex value of the base structure of boron sheets is listed in Table 5.

**Table 5:** Experimental data for Young’s modulus and shear modulus of boron nanosheets.

SI. no	Boron nanosheets	Shear modulus, *G*	Young’s modulus, *E*
1	$\alpha$-icosahedral, $\alpha$-$B_{12}$	210 GPa	480 N/m
2	$\alpha$ borophene	88 GPa	210 N/m
3	$8-Pmmn$ borophene	108 GPa	241 N/m
4	$\beta _{12}-$borphene	68.5 GPa	179 N/m

In our study, we investigate the elastic, geometric, thermodynamic, and mechanical properties of the boron sheets. An elastic constant is used to determine the mechanical properties of a material and describe its ability to resist deformation by external forces. With elastic constant, some mechanical properties such as Young’s modulus *E*, bulk modulus *B* and Shear modulus *G* can be determined. The elastic properties are closely related to the thermodynamic properties like melting point, heat capacity, vacancy defect, and temperature. The Young’s modulus, *E*, and Shear modulus, *G* data of various boron nanosheets are summarized in Table 6^44–46^. The Young’s modulus (N/m) indicates a material’s ability to withstand changes in length when brought under tension or compression and shear modulus (GPa) is a measure of elastic shear material’s stiffness that reflects body rigidity.

**Table 6:** Degree-vertex value of boron nanosheets.

Indices	$\alpha$-icosahedral	$\alpha$-borophene	$8-pmmn$	$\beta _{12}$-borophene
$M_1$	3140	1222	1364	1318
$M_2$	8522	2968	3441	3023
$M_2^m$	10.08	6.1511	6.0986	7.6156
*A*	10637.932	3520.5917	3910.979	3508.5186
*R*	8522	2968	3441	3028
*RR*	10.08	6.1511	6.0986	7.6156
*H*	53.8545	26.849	26.1612	31.9423
*HM*	34184	12148	14714	12452
*F*	17140	6212	7832	6814
$\sigma$	96	276	950	360
*SDD*	583.2	266.667	313.9048	304.9333
*I*	782.8182	297.9432	316.349	319.6294

### Properties analysis and theoretical prediction

The mechanical properties, Young’s modulus and shear modulus of the above-mentioned boron sheets are analyzed with topological descriptors by a regression model. Legendre^47^ and Gauss^48^ introduced the least squares approach to linear regression in 1805 and 1809 respectively. Regression analysis is a statistical technique that determines the correlation between two or more variables. The correlation coefficient ranges from 1 to -1. The perfect positive and negative correlation is 1 and -1 where near 0 indicates weak correlation. A correlation coefficient and regression analysis are used to derive the equation connecting the descriptors and properties. The linear regression model,

$$\begin{aligned} M=i+j(TD) \end{aligned}$$

where M is the mechanical properties of the boron nanosheets, and TD is topological descriptors. Using SPSS software^49,50^, the invariant, *i* and regression coefficient, *j* can be calculated. The correlation coefficients between dependent variables, physical properties of boron sheets and independent variables, topological descriptors of nanosheets are listed in Table 7. For recent work on QSPR analysis by linear regression method, readers can refer^51,52^.

**Figure 10:**
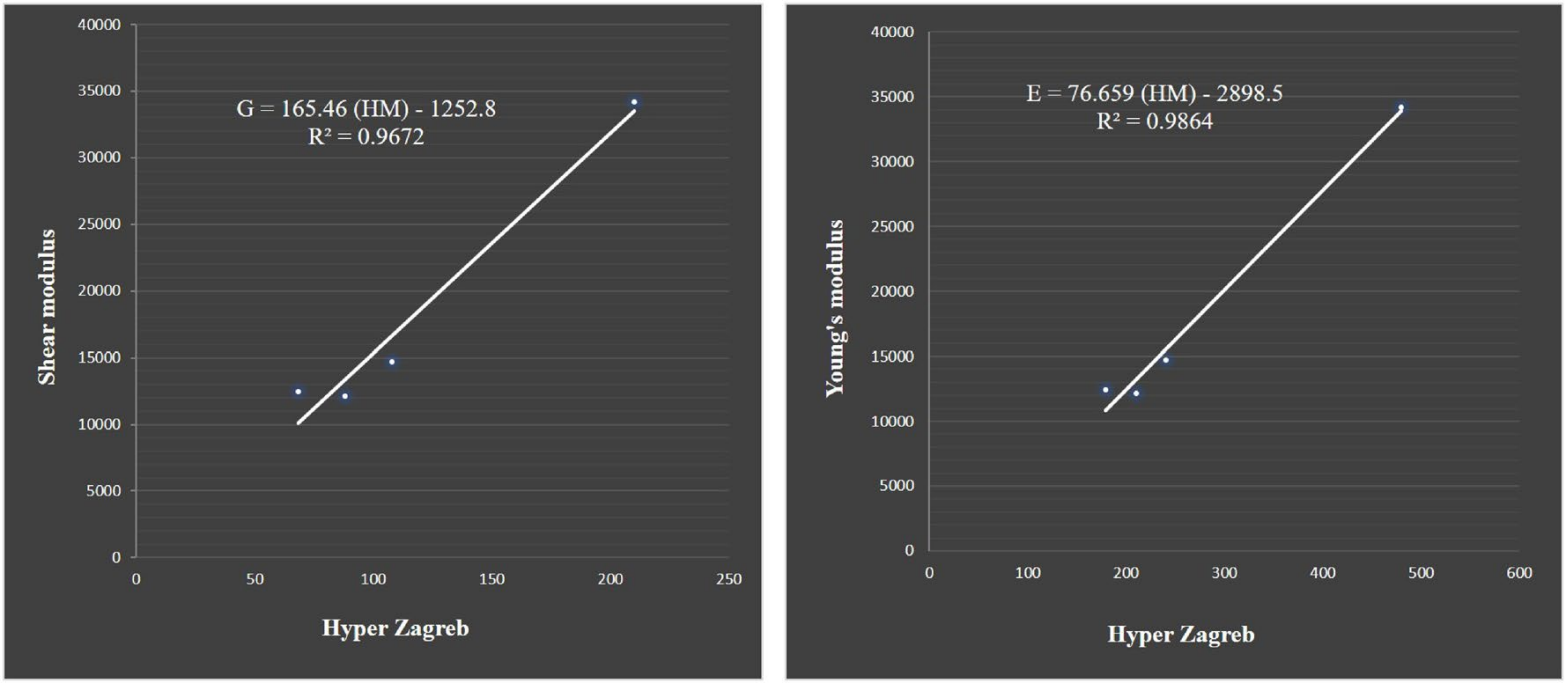
Scatter visualisation for the properties and indices.

The correlation table indicates that these boron derivatives have strong correlations within themselves for both chemical attributes. In comparison to other indices, the hyper Zagreb index has a strong correlation for Young’s modulus and shear modulus. The linear regression model for shear modulus is shown below,

$$\begin{aligned} G=165.46(HM)-1252.8 \end{aligned}$$

where G is shear modulus and HM is hyper Zagreb index. Similarly, the linear regression equation for Young’s modulus is determined as follows

$$\begin{aligned} E= 76.659 (HM)-2898.5 \end{aligned}$$

where E is Young’s modulus. The molecular characteristics with a greater dimension can be predicted with an appropriate regression model. In Fig. 10, the scatter plots for the highest correlated properties and descriptors are shown.

**Table 7:** Correlation coefficient between properties and descriptors.

Indices	Shear modulus, *G*	Young’s modulus, *E*
$M_1$	0.9701	0.981
$M_2$	0.98	0.9915
$M_2^m$	0.8109	0.8494
*A*	0.9774	0.9901
*R*	0.9798	0.9914
*RR*	0.8109	0.8494
*H*	0.9013	0.93005
*HM*	0.9835	0.9932
*F*	0.9794	0.989
$\sigma$	0.39799	0.45497
*SDD*	0.9636	0.9777
*I*	0.9645	0.98102

## Conclusion

Using a degree-vertex M-polynomial graph technique, the topological indices of the boron $\alpha$-icosahedral nanosheet are determined. The structural characterization is used to analyse the topological connectivity properties of boron $\alpha$-icosahedral nanosheet, by combining quantum chemical descriptors with nanosheet results. This research could provide a crucial tool for determining the significance of nanosheets in many areas, such as material science, drug discovery, and predictive toxicology. Furthermore, the topological indices are used in the study of boron $\alpha$-icosahedral nanosheets and provide QSAR expressions that predict several molecular properties such as band gap, optical and electronic stability, molecular density, enthalpies, conductivity, and so on. In this research, we correlate our theoretical results with the shear modulus and Young’s modulus original data synthesized in recent years, which showed a high correlation of 0.9835 and 0.9932 with hyper Zagreb. This type of research has not been explored earlier. So, it has a significant contribution to research by finding a correlation between topological indices and properties of boron allotropes. This allows us to explore other nanosheets, it is left as an open problem for future research.

## Data Availability

The datasets generated and/or analyz during the current study are available in this current article.
